# An integrated analysis of miRNA and mRNA expressions in soybean response to boron stress

**DOI:** 10.1371/journal.pone.0328882

**Published:** 2025-07-28

**Authors:** Qiang Li, Jiahua Guo, Xuejiao Wang, Xiaoyu Zhao, Kunyu Liu, Xiaohui Feng, Wei Liu, Xuefei Gu, Youwei Che, Guangping Chen, Jianjun Yao, Lijun Li, Yuanli Gao, Erhu Su

**Affiliations:** 1 Inner Mongolia Academy of Agricultural & Animal Husbandry Sciences, Hohhot, Inner Mongolia, China; 2 Baotou Teachers’ College, Baotou, Inner Mongolia, China; 3 Oroqen Autonomous Banner Agriculture Stockbreeding Science and Technology Career Development Center, Hulun Buir, Inner Mongolia, China; 4 Daur Autonomous Banner of Morin Dawa Agriculture Technology Popularizing Center, Hulun Buir, Inner Mongolia, China; 5 Inner Mongolia Autonomous Region Agriculture and Animal Husbandry Technology Promotion Center, Hohhot, Inner Mongolia, China; Dokuz Eylul Universitesi, TÜRKIYE

## Abstract

Boron (B) fertilization significantly increased soybean seed yields and is an indispensable micronutrient in soybean growth. However, it is difficult to absorb in soil, which can seriously affect soybean growth. In this study, soybeans were planted under B deficiency and B sufficiency conditions, then physiological and biochemical indicators of soybean seedlings were measured. The results indicated that the antioxidant enzyme activity (SOD, POD, CAT, APX) and malondialdehyde content increased considerably compared with their controls in the leaves and roots, respectively. Among these, the difference in the roots was more significant than in the leaves, suggesting that the root system was more sensitive. Therefore, soybean roots under B stress for 12 hours and 8 days were selected to construct the miRNA library for sequencing. In the three comparison groups, 55 miRNAs were differentially expressed in response to B stress, 22 known miRNAs, and 33 novel miRNAs were identified. Through mRNA-miRNA meta-analysis, miRNA-objective gene pairs consisting of 21 DEGs and 11 miRNAs were identified. The GO functional annotation indicated that stress response genes were mostly concentrated in the items of enzyme activity, ion transport, and metabolic processes, etc. In KEGG of 8d, the objective genes were drastically enriched cyanoamino acid metabolism, glycine, serine and threonine metabolism, phenylalanine metabolism, etc. We further constructed the pathway for cyanide amino acid metabolism under B stress and found that miR408c-5p targeted the gene controlling β -glucosidase 18, and the expression level was notably decreased. It was speculated that B stress hindered soybean growth by inhibiting amino acid metabolism and affecting other metabolic pathways. This study combined miRNAs and mRNAs to identify DEMs and metabolic pathways correlated to B stress. This study provides information that will help elucidate the complex mechanism of the B stress response in soybeans. Moreover, candidate miRNAs and mRNAs could yield new strategies for the development of B-tolerant soybean breeding.

## Introduction

Soybeans (*Glycine max* [L.] Merr.) area type of legume widely cultivated worldwide, because they have a wide range of uses and significant economic significance. They are a prominent source of protein and edible oil and have valuable uses as food, feed, and oilseed crop [1]. Therefore, it is essential to increase the yield of soybeans. In 2022, the global average soybean yield was 182.67 kg/ha. Despite the cultivation of many excellent varieties by cultivators, crop yields remain very low. Micronutrient deficiency may be a significant contributing factor [2].

Micronutrients viz., iron (Fe), manganese (Mn), zinc (Zn), copper (Cu), boron (B), molybdenum (Mo), chlorine (Cl) and nickel (Ni) are absorbed by plants in a small proportion, but they are just as important as constant nutrients in production practice [3]. B positively participates in the growth and development of crops. The regulation of protective enzyme system, cell wall synthesis, sugar transport, fission, differentiation, membrane function, root extension, Ca^2+^/calmodulin system, signaling, phytohormone and so on are closely associated [4,5]. According to Furlani et al. [6], soybeans are among the crops with the highest demand for boron, but often experience insufficient quantities. Boron deficiency can lead to yellowing of young leaves and chlorosis between leaf veins [1] and break the balance of reactive oxygen species in plants [7].Researchers reported that B treatments substantially influenced the plant height, pods per plant and seed yield [8]. Boron stress in plants has been studied in a variety of ways, including mRNAs, lncRNAs, miRNAs, and so on [9–11]. In addition, a whole transcriptome characterization of R2R3-MYB transcription factors has been performed [12].

Microribonucleic acid (miRNA) is a fundamental endogenous small ribonucleic acid, widely distributed in plants and a key regulatory molecule in response to plant stress. Its main components exhibit high conservatism between species [13,14]. An increasing body suggests that plant miRNA regulation is crucial for functional genes involved in its development, embracing morphogenesis, meristematic tissue structure, and responses to biological and environmental stresses [15,16]. For example, miR395, miR398, and miR399 respond to sulfur (S), copper (Cu), and Pi deficiency, respectively [17–19], while miR169 participates in the root development [20]. However, the exact involvement of miRNA in the mechanism of soybean boron reaction remains uncertain, indicating the need for further research.

In this study, MK9 (Mengkedou No. 9 (MK9) is a new soybean variety independently selected and bred by the Inner Mongolia Academy of Agricultural & Animal Husbandry Sciences) was used as the experimental material, and the physiological indicators of soybean roots and leaves were measured at different time points under B stress. Soybean root samples are sensitive to B, which were subjected to high-throughput sequencing and bioinformatics analysis. Based on high-throughput sequencing results, we analyzed the differentially expressed microRNAs (DEMs) in soybeans and investigated the enrichment pathways of GO and KEGG under B stress. Then combined with mRNA, to study the metabolic pathways and molecular networks of objective genes connected to B stress. Meanwhile, qRT-PCR validated their expression levels. The aim was to understand the response mechanism of B stress in soybean and to establish a theoretical foundation for crop improvement and stress resistance breeding.

## Materials & methods

### Preparation of plant materials

Soybean (*Glycine max* [L.] Merr.)variety MK9 was used, and was collected from Inner Mongolia Academy of Agricultural & Animal Husbandry Sciences (40°51' N, 110°46' E). First, the seeds were placed in a 10% NaClO solution and disinfected for 10 minutes. Then, the seeds were washed with distilled water and soaked for 4 hours. Finally, the seeds were cultured and sprouted under normal temperature, under dark and humid conditions. Four days later, the sprouted seeds were cultured in a 16-L water tank with half concentration of modified Hoagland nutrient solution [21]. The nutrient solution for cultivating plants was replaced every two days. The light/dark cultivation cycle of the incubator was set to 16/8 hours, with a light/dark temperature of 26/22 °C and a optical power of 150 μmol m^-2^ s^-1^ [22]. After 8 days, the soybeans were transferred to a control solution (containing H_3_BO_3_) and B deficient solution (excluding H_3_BO_3_), respectively. The response of plants was slow after the stress of micronutrients. Based on previous studies, we also sampled the roots and leaves of soybean seedlings after 12 hours, 24 hours, 72 hours, and 8 days of cultivation, respectively. Then the samples were frozen in liquid nitrogen, which were stored in a refrigerator at −80 °C for subsequent testing. We named T1, T2, T3 and T4 after 12h, 24h, 72h and 8d of B stress treatment, respectively, and the corresponding controls were named CK1, CK2, CK3 and CK4. The control roots and leaves were labelled CK-R and CK-L, respectively; the B deficiency treatments of the roots and leaves were labelled T-R and T-L, respectively. We replicated three sets.

### Determination of protective enzyme system

We ground the frozen root sample with liquid nitrogen, weigh 0.1g, and place in a 1.5mL centrifuge tube. Added extracts separately, and used a limited company reagent kit to determine the superoxide dismutase (SOD), peroxidase (POD), catalase (CAT) and ascorbate oxidase (APX) activity, as well as the malondialdehyde (MDA) content [9].

### Small RNA and mRNA library construction and sequencing

We used the Trizol reagent (Invitrogen Life Technologies) to extract RNA. Then used a NanoDrop spectrophotometer (Thermo Scientific) to measure concentration, mass, and integrity. The transcriptomic sequencing results refer to Jiahua Guo’s experimental protocol [9]. According to the instructions, we constructed a small RNA library using the NEBNext multiplex small RNA library preparation set for Illumina (New England Biolabs, Inc.). In summary, we used a ligase mixture to 1 μg of total RNA from the soybean root samples (CK1, T1, CK4, T4, respectively), which was connected to 3 ‘and 5’ adapters. The obtained samples were reverse transcribed (Superscript II reverse transcriptase). We conducted PCR product amplification. Small RNA libraries were analyzed for QC and average size of inserts was approximately 140–150 bp. We used the Bioanalyzer 2100 system (Agilent) and NovaSeq 6000 platform (Illumina) to quantify and sequence the sequencing library.

### Acquisition of miRNAs

The quality information of raw data in FASTQ format was calculated, and then the raw data was filtered using a script developed by Personalbio company. Clean data was obtained by removing adapters and Low-quality sequence. Filter Clean Reads from 18 nt to 36 nt in length and performed deduplication to obtain Unique Reads for subsequent analysis. The reference genome index was constructed by Bowtie2 (v2.2.6) and mapped to the reference genome using miRDeep2 (v2.0.0.8) software.

First, unique Reads in the miRBase database (http://www.mirbase.org/) was annotated with known miRNAs and then annotated with other non-coding RNAs. Sequences were not annotated with any information using mireap(v2.0) for new miRNA prediction analysis.

### Screening of differentially expressed miRNAs and prediction of objective genes

We used DESeq (v1.30.0) to screen for differentially expressed genes, a threshold set to |log2FoldChange | > 1 and P-value<0.05. MiRanda (v3.3a) was used to predict objective genes for differentially expressed miRNA sequences.

### GO and KEGG

We performed Gene ontology (GO, http://geneontology.org/) enrichment analysis on the objective genes of differential miRNA using top GO. The P-value was calculated using the hypergeometric distribution method (the standard of significant enrichment is P-value < 0.05). The cluster Profiler (3.4.4) software was used to carry out the enrichment analysis of the Kyoto Encyclopedia of Genes and Genomes (KEGG, http://www.kegg.jp/) pathway of the objective genes of differential miRNA. We mainly focused on significant enrichment pathways with P-value<0.05.

### qRT-PCR analysis

To test the accuracy of miRNA sequencing data, we picked five essential miRNAs for qRT-PCR. We chose miR156a as the internal reference. We used 2^-ΔΔCt^ calculation of relative expression level. We also replicated three sets. P < 0.05 is considered to have a significant difference.

## Results

### Determination of physiological indexes of soybean seedlings

Malondialdehyde (MDA), a byproduct of membrane lipid peroxidation, is known to exacerbate membrane damage. The antioxidant enzyme system plays a crucial role in eliminating free radicals within cells, thereby maintaining the oxidation-reduction balance.

**Fig 1:**
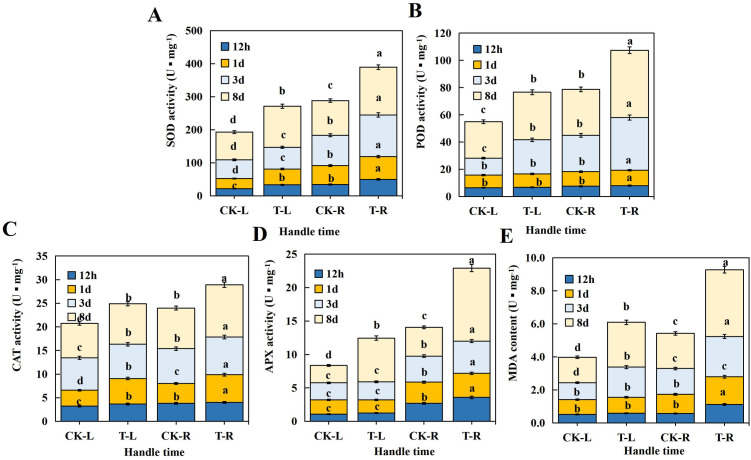
(A) Activity of SOD under B stress; (B) Activity of POD under B stress; (C) Activity of CAT under B stress; (D) Activity of APX under B stress; (E) Content of MDA under B stress activity. CK-L: the leaves of soybean seedlings under B-sufficiency; T-L: the leaves of soybean seedlings under B-deficiency; CK-R: the roots of soybean seedlings under B-sufficiency; T-R: the roots of soybean seedlings under B-deficiency. The lowercase letters represent values within p < 0.05. Note: Upper case letters in the figure indicate the name of the picture and lower case letters indicate the analysis of significance.

Determined the physiological indexes of the leaves and roots of soybean seedlings at four time points (12h, 24h, 72h and 8d), respectively, after B stress. The activity of antioxidant enzyme system and MDA content in soybean were always higher than those in the contrast (Fig 1). The MDA content in leaves and roots increased substantially with the increase of stress time, and compared with the control, it increased significantly in the roots. The MDA content reached the highest point at 8 days (Fig 1E), indicating that long-term B stress seriously damaged cell membrane of the soybeans.

The activities of four antioxidant enzymes, SOD, POD, CAT and APX (Fig 1A–D), showed an upward trend with the increase of stress time. In 8d, the activities of four antioxidant enzymes reached the highest point, indicating that with increase of stress time, antioxidant defense system in the soybeans was seriously lesioned and the antioxidant enzymes hindered the normal growth and metabolism of the soybeans.

However, at 12h, 24h and 72h, the POD, APX activity and the MDA content did not show significant differences compared to the control. It was not until 8d that the physiological indexes exhibited extremely significant differences from those of the control, with the differences in the roots being notably higher than those in the leaves.

It shows that the physiological index of soybeans need a certain reaction time under B stress, were most affected at 8d, and the roots were more susceptible than the foliages with B stress. Therefore, root samples of soybeans at 12h and 8d were selected for high-throughput sequencing of miRNA.

### High-Throughput sequencing of miRNAs from soybean

In order to further analyze the molecular response mechanism of soybeans to B stress, soybean roots at 12h and 8d were sequenced (high-throughput miRNA sequencing)under B stress, respectively. In this study, the average raw data obtained from CK1 and CK4 (B-sufficiency), T1 and T4 (B-deficiency) were 22 519 66 and 17 426 395, 21 917 217 and 19 544 943, respectively. After removing the junk sequences, the clean reads with the length of 18nt-36nt were collected for further analysis, and the clean reads of 10 484 526 and 9 888 581, 10 952 575 and 11 596 652 were obtained from CK1 and CK4 and T1 and T4, respectively (Table 1). Data observation shows that the abundances of miRNAs in the libraries increased after B stress treatment, with T1 and T4 increasing by 4.3% and 14.73% respectively, compared with the CK. This evidence indicates that B stress may increase the expression level of miRNA in soybeans, while T4 showed greater changes in miRNAs due to a longer treatment time.

**Table 1:** The number of reads for soybean miRNA sequencing and comparison.

Length	raw reads	clean reads
CK1_1	20 892 881	10 374 748
CK1_2	21 678 244	10 124 497
CK1_3	24 987 877	10 954 332
CK4_1	17 246 624	9 409 809
CK4_2	19 073 209	11595958
CK4_3	15 959 353	8 659 976
T1_1	22 258 431	8 714 602
T1_2	19 095 213	10 888 069
T1_3	24 398 007	13 255 053
T4_1	17 579 200	10 116 020
T4_2	15 797 188	10 432 009
T4_3	25 258 440	14 241 928

**Fig 2:**
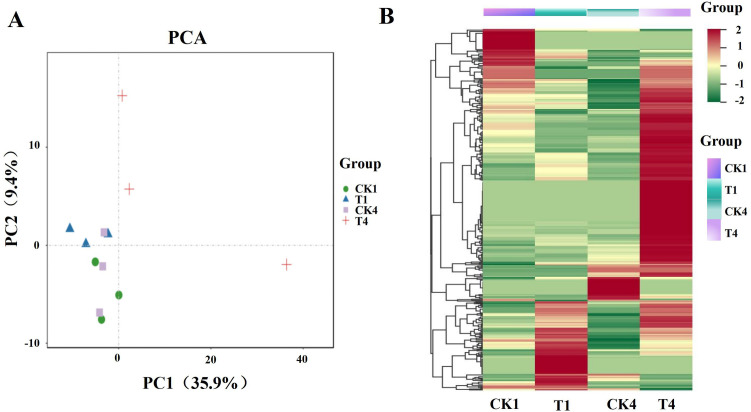
The miRNA analysis of soybean. (A) PCA of each group of samples; (B) Number of expression miRNA in each group of samples.

PCA results showed (Fig 2A) that the contribution of the first principal component (PC1) was 35.9%. With the prolongation of boron stress, there was a significant difference between T4 and other samples. This was further corroborated by the data from the heat map (Fig 2B), where miRNAs were significantly up-regulated in expression at the T4 stage. This may be due to the great influence of boron stress on the plants, which fully justifies the necessity of this study.

### Differential expression of miRNAs (DEMs) under B stress at different stages

**Fig 3:**
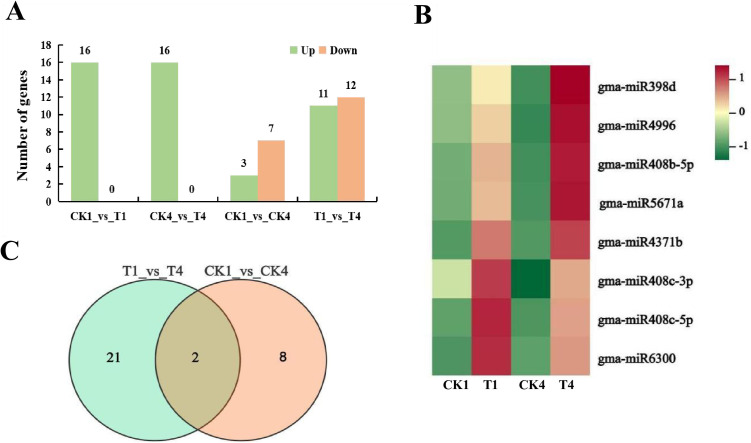
(A) DEMs in different comparison groups under boron stress at different time; (B) Heat map of 8 DEMs according to logarithmic transformations of normalized reads. Common DEMs in CK1_vs_T1 and CK4_vs_T4. (C) Number of DEGs in CK1_vs_CK4 and T1_vs_T4.

Differentially expressed miRNAs (DEMs) were screened from the above identified to miRNAs. 65 significant differentially expressed miRNAs were found from four comparative combinations. CK1_vs_T1 and CK4_vs_T4 mainly analyzed the DEMs after B stress at different times while CK1_vs_CK4 and T1_vs_T4 mainly analyzed the DEMs of soybean seedlings in B deficiency for a long time. The results showed that there were 55 DEMs in the three comparison groups (CK1_vs_T1, CK4_vs_T4 and T1_vs_T4). There were considerably more up-regulated than down-regulated of genes in CK1_vs_T1 and CK4_vs_T4 (Fig 3A). Of the 55 DEMs, 22 conserved miRNAs (40%) and 33 non-conserved miRNAs (60%). This shows that specific miRNAs may be crucial for B stress response.

Compared to the control, after 12h and 8d of B stress, there were 8 DEMs in the two comparison groups (CK1_vs_T1 and CK4_vs_T4). It was observed that all of them were up-regulated according the Heatmap. Specifically, gma-miR408c-5p, gma-miR408c-3p, gma-miR6300 of B stress showed strong upregulation at 12h, while the remaining 5 microRNAs gma-miR4371b, gma-miR408b-5p, gma-miR5671a, gma-miR398d, gma-miR4996 were strongly upregulated at 8 days of B stress (Fig 3B). This finding suggests that as the duration of B stress increases, the expression levels of differentially expressed microRNAs (DEMs) rise extensively compared to the CK, indicating a heightened response to B stress.

Compared to B stress for 12h, after 8d there were 10 DEMs with B-sufficiency, of which 3 up-regulated and 7 were down-regulated; there were 23 DEMs with B deficiency, of which 11 up-regulated genes and 12 down-regulated in soybean seedings. Combining the analysis of the two comparison groups, we found that there were two common differentially expressed DEMs in T1_vs_T4 and CK1_vs_CK4 (Fig 3C): gma-miR169n-5p and gma-miR408c-3p. These DEMs may be linked to soybean growth, as they were significantly down-regulated. However, the extent of down-regulation differed, possibly due to the influence of B stress.

The gma-miR6300 was found in CK1_vs_T1, CK4_vs_T4 and T1_vs_T4, which indicated that it was closely associated with the growth inhibition of the soybeans under B deficiency, and its objective gene GLYMA_04G055400 was drastically down-regulated in the mRNA. This gene is related to quinone oxidoreductase subunit in chloroplasts, indicating that B stress may further inhibit the growth of chloroplasts in soybean seedlings by affecting their synthesis, which can be further studied.

### GO function annotation and pathway analysis of the DEMs

Among the differential miRNAs identified above, 43 miRNAs were predicted to have 695 target genes, of which 779 target sites. GO function annotation analysis were found that CK1_vs_T1 contains 195 GO iterms, and CK4_vs_T4 contains 227 GO iterms. These results indicate that the inhibitory effect on soybean seedlings significantly increases with the duration of B stress.

**Fig 4:**
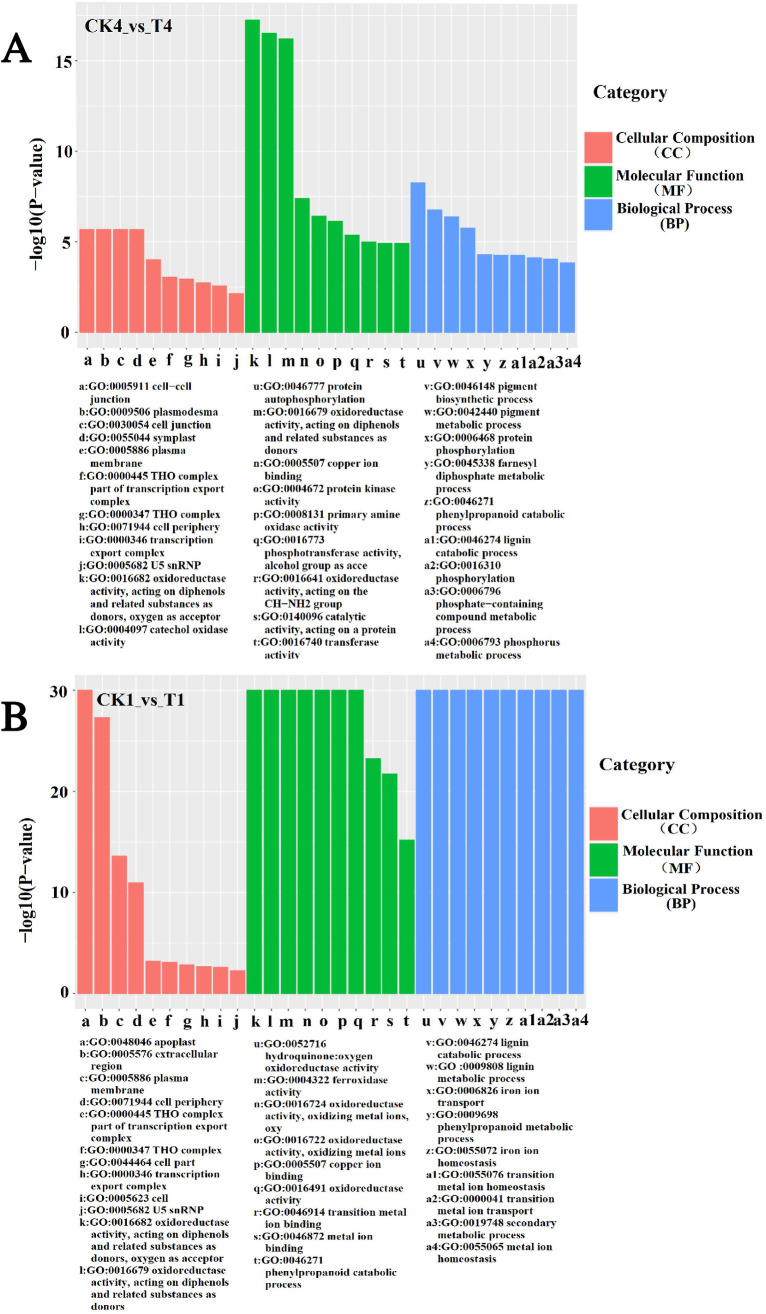
GO function annotations of target genes of DEMs in soybean genotypes of (A) CK1_vs_T1 and (B) CK4_vs_T4.

In the cellular composition (CC), they were significantly enriched in extracellular areas, extracellular perimeter, etc., at 12 hours of boron stress. When boron stress 8 days, they were significantly enriched in inter -cytoplasm, cell connection, etc. These results indicate that the effects of boron on the cellular components of the plants in the pre-stress period were mainly focused on the cellular periphery, etc., and that the boron deficiency caused damage to the cellular structure with the extension of the stress time, which were consistent with the transcriptome. In the molecular function (MF), they were significantly enriched in enzyme activity and ion transport, suggesting that boron stress has an effect on plant metabolism, possibly accelerating plant senescence, and possibly on the transport and binding of other ions as well at both time points, which are consistent with the transcriptome. Specifically speaking, gma-miR5770a- GLYMA_20G145100 was related to oxidase. In the biological process (BP), at two time points, these genes had a high ratio in metabolic processes, indicating that boron deficiency may affect plant metabolism and hinder plant growth and development, together with their ability to respond to abiotic stress in the environment. These findings are consistent with transcriptomics (Fig 4). There were four groups of miRNA-mRNA in CC, MF and BP: gma-miR4996-GLYMA_10G239000, gma-miR5770a-GLYMA_03G005200, gma-miR408c-3p-GLYMA_07G115000, gma-miR408c-3p-GLYMA_08G128100 and indicated that the four miRNA-mRNA may all be correlated with the soybean growth and metabolism under the B stress, among which the GLYMA_10G239000 was related to proteins.

**Fig 5:**
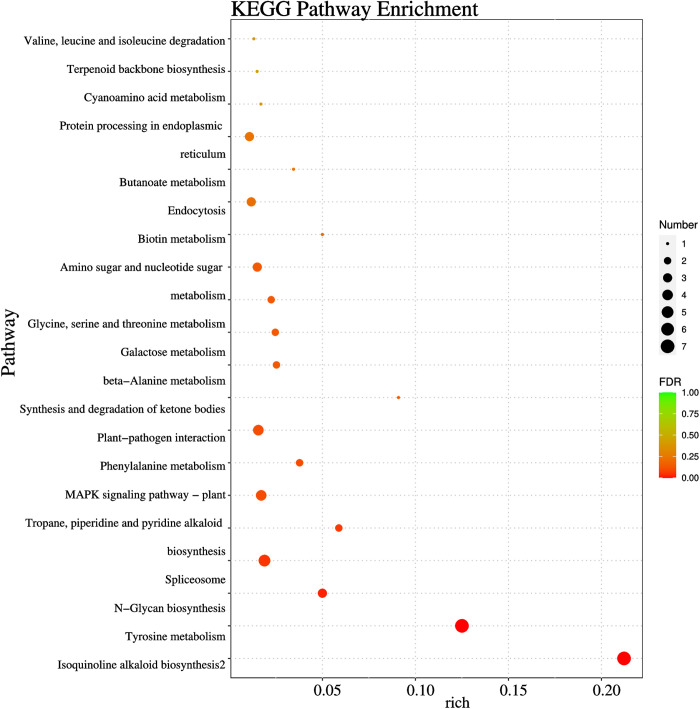
KEGG pathway enrichment analysis of target genes of DEMs in CK4_vs_T4.

In the KEGG analysis, the stress time of T4 treatment was the longest (8d), so the pathways of CK4_ Vs_ T4 was more abundant and significantly enriched to a total of 23 metabolic pathways (Fig 5). Among them, cyanoamino acid metabolism, glycine, serine and threonine metabolism, phenylalanine metabolism, etc. have a significant increased in the transcription group results. These metabolisms may act an important part in plant response to the boron coercion process. The KEGG pathway contained a targeting relationship, gma-miR5770a-GLYMA_ 20G145100, was also in GO enrichment of MF, which was mainly associated with soybean enzyme activity and metabolism. From the above results, we speculated that B stress mainly affects the metabolic pathway of soybeans, thus inhibiting plant growth.

### Involvement of miRNAs in B stress of soybean

**Fig 6:**
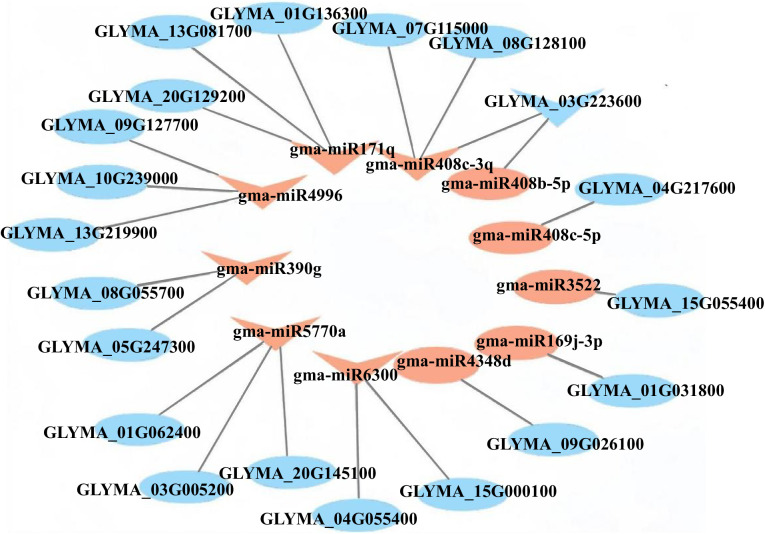
Potential miRNA mRNA regulatory modules are related to boron stress in soybean.

The same miRNA could target different genes, and their functions were similar (Fig 6). Under the influence of boron stress, these miRNAs are upregulated and inhibit the expression of target genes, resulting in a series of effects on the plant. The gma-miR171q targeted genes were all linked to functional proteins (GLYMA_01G136300, GLYMA_13G081700, GLYMA_15G055400). The gma-miR408 family targeted genes were connected to protein RADIALIS-like 3 (GLYMA_03G223600, GLYMA_03G223600, GLYMA_07G115000, GLYMA_08G128100, GLYMA_04G217600). The gma-miR4996 targeted genes were related to material transport pathways (GLYMA_09G127700, GLYMA_10G239000, GLYMA_13G219900). The gma-miR5770a targeted genes were associated with (RefSeq) primary amine oxidase (GLYMA_20G145100). The gma-miR6300 targeted genes were associated with NAD (p) h-quinone oxidase subunit U (GLYMA_04G055400) and linked to 65-kda microtubule (GLYMA_15G000100), and it is speculated that it may affect the formation of membrane bodies on chloroplasts. In addition, the gma-miR169j-3p targeted gene influences potassium transporter (GLYMA_01G031800). The gma-miR4348d targeted gene was related to tubulin beta chain (GLYMA_09G026100).

Based on the analysis of 21 objective gene pairs, it could be inferred that B stress mainly affected the material transport, oxidase activity, photosynthesis, and functional proteins in soybean seedlings. In the next stage, studying these target genes will help to better understand the regulatory role of miRNA in plants facing B stress.

Future studies on these objective gene pairs will facilitate a better understanding of the regulatory role of miRNA in plants facing B stress.

### Quantitative PCR expression analysis

**Fig 7:**
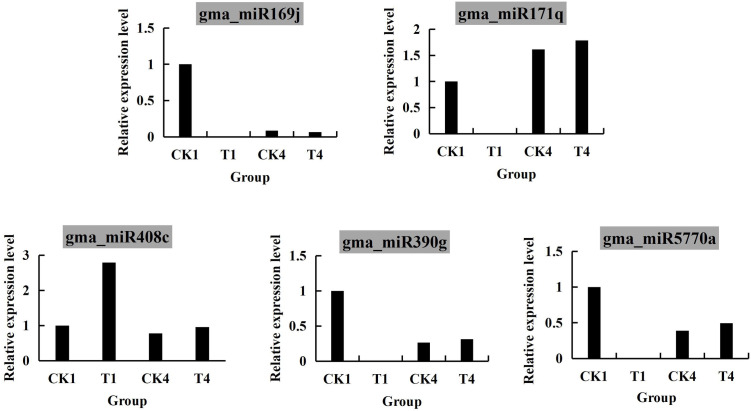
Expression analysis of miRNAs by qRT-PCR.

To verify the dependability of miRNA results, 5 miRNAs were selected from four comparative groups for qRT-PCR analysis. After research, it was found that the qRT-PCR were identify with the miRNA sequencing results, which indicated that the miRNA sequencing was trustworthy in this study (Fig 7, S1 Table). Among them, gma_ miR408c-3p was present in all four comparative groups, indicating that this miRNA may be associated with soybean growth and metabolism; Both CK1 and CK4 soybean seedlings grew in an environment with B-sufficiency, while the only difference between them was the growth time. Therefore, in CK1_vs_CK4 DEMs, gma_ mMiR5770a, gma_ miR390g and gma_miR169j-3p may be related to soybean growth and the difference was significant, while gma_MiR5770a in CK4_ Vs_ T4 also showed significant differences, indicating that this miRNA may be linked to B stress in soybean; At T1_ Vs_ T4, gma_miR171q was extensively upregulated, indicating that with the increase of B-deficiency time, the effect of the objective gene corresponding to gma_miR171q was gradually increasing, which may inhibit soybean growth.

## Discussion

B is a crucial factor in soybean growth. Hari Ram et al. reported that B treatments notably influenced the soybean height, pods per plant and seed yield [8]. However, research on how to regulate soybean growth with B deficiency is very rare. It is crucial for researchers to study miRNAs under abiotic stress [23,24]. To explore the role of miRNA in B stress, four libraries were constructed for different time periods (12h, 8d)of B treatment. In our current study, 55 DEMs were identified, including 22 conserved miRNAs and 33 non-conserved miRNAs. There are more non-conserved miRNAs than conserved miRNAs, which is consistent with the research results of lower quantity and expression of conserved miRNAs in wild soybeans [25]. These results further confirmed that non-conserved minRNAs may develop pivotal role in the universal regulatory mechanisms of plants.

Lack of protection does not represent loss of functionality [26]. Gma-miR408c-3p, gma-miR6300, gma-miR169j-3p, miR396d, gma-miR398b-5p, and gma-miR371b are non-conserved miRNAs, but they are Leguminosae specific. Researchers have shown that conserved miRNAs may promote transcriptional regulation related genes, while non-conserved miRNAs may act a crucial role in regulating the inherent bioprocess of Leguminosae [27]. In this study, after 12h and 8d of B stress, there were 22 non-conserved miRNAs and 12 conserved miRNAs, among which the objective genes corresponding to non-conserved miRNAs were mostly linked to glycosyltransferase, amino acid metabolism and catalytic activity. The objective genes corresponding to conserved miRNAs were mostly associated with internal proteins, which aligns with previous research findings.

Chloroplast is very important for plant growth. If the synthesis is blocked, the veins will turn yellow, or even the growing point of soybean will die, which will inhibit plant growth [1]. In our experiment, miR6300was extensively up-regulated after 8d of B stress in soybean, and it was found in three comparison groups, CK1_vs_T1, CK4_vs_T4 and T1_vs_T4, and its objective gene GLYMA_04G055400 encoded the NAD(P)H-quinone oxidoreductase subunit U. In the electron transfer chain, it is the largest and most complex enzyme, and its down regulation can affect the electron transfer process, leading to the accumulation of oxygen atoms and chlorophyll degradation, thereby affecting photosynthesis [28].

**Fig 8:**
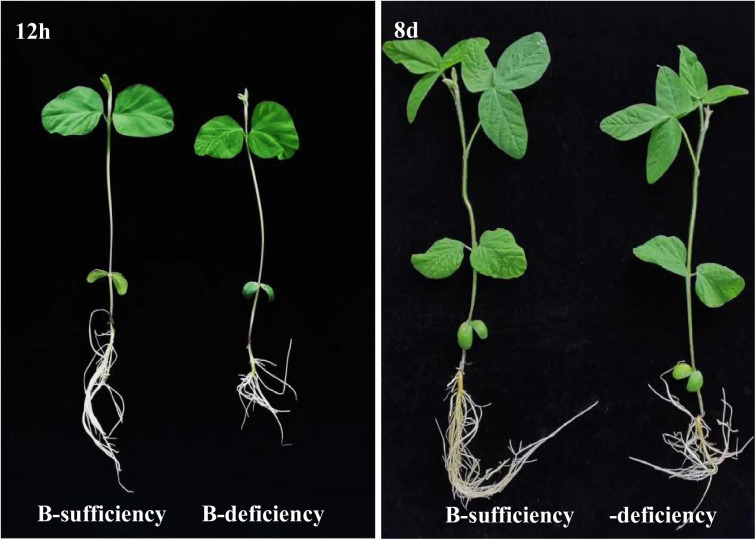
The soybean seedlings under B-deficiency (without 0.045mM H_3_BO_3_) and B-sufficiency (with 0.045mM H_3_BO_3_) conditions, which were cultivated for 12h and 8d under hydroponic culture systems [9].

Our research group has measured the SPAD value of relative chlorophyll content of soybean seedlings under B deficiency and B sufficiency at 12h and 8d, respectively. We found that the SPAD value was always significantly lower than that B sufficiency in different times, indicating that B stress will affect the synthesis of soybean chlorophyll and we can directly observe soybean seedling growth (Fig 8), finding that the B stress notably inhibits soybean growth [9]. This research result is consistent with our findings. Next, we can further explore the targeting impact of miR6300 to GLYMA_ 04G055400.

**Fig 9:**
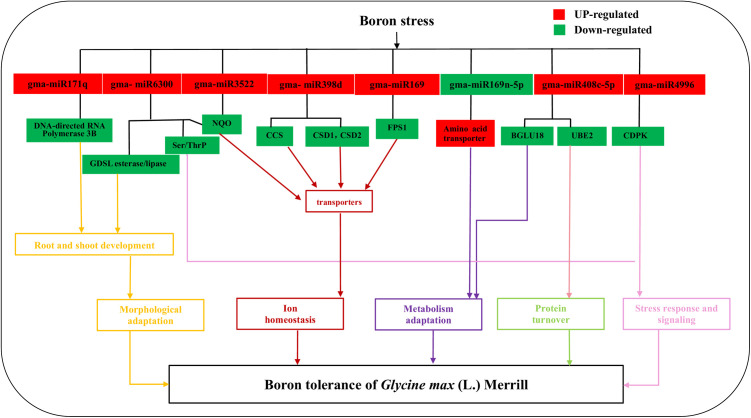
Potential regulatory network of boron stress miRNAs in *Glycine max* (L. **) Merrill.** NQO: NAD(P)H-quinone oxidoreductase subunit, chloroplasts; CCS: Cu/Zn-superoxide dismutases; FPS1: farnesyl diphosphate synthase isoform 1S; PPO: polyphenol oxidase; CDPK: calcium dependent protein kinase; NADP-ME: NADP-dependent malic enzyme; BGLU18: beta-glucosidase 18.

The ability of the root system, as the main organ for plants to obtain nutrients from the soil, depends on its physiology and morphology [29,30]. In past study, we found that B deficiency would inhibit the growth and development of soybean seedling roots (Fig 8). In this study, the DEMs of miR6300 targeted control of GATA2 and GDSL esterase/lipase (Fig 9), in which GATA transcription factors were bound with root growth and development and its response to abiotic stress [31–33]; GDSL esterase/lipase is broadly-based participation in plant growth and development, secondary metabolism and stress [34]. In this research, the Significantly upregulated of miR6300 may affect the functional activities of GATA2 and GDSL esterase/lipase and inhibit the root growth of soybean seedlings. MiR396 are highly conserved in diverse plant species and widely distributed in similar regulatory pathways in different species [35]. The analysis of Lotus mRNA and Small RNA Sequencing showed that down-regulation of miR396a and miR396b can prevent the synthesis of amylopectin and total starch while in rice [23], over-expression of miR396c owered salt-alkalia tolerance [36]. MiR396 is upregulated in Arabidopsis, leading to inhibition of growth regulatory factor (GRF) and subsequent inhibition of cell proliferation [37]. In this study, the expression of miR396 increased sharply with 12h of B stress, and the difference was extremely significant, indicating that miR396 might be related to B stress in soybeans. By controlling GRF, it inhibited the proliferation of soybean root cells, and further inhibited soybean root growth. In the comparison figure of soybean grew with B sufficiency and B deficiency (Fig 8), it also proved that the growth of soybean roots was suppressed under B stress.

Some studies have shown that under abiotic stress such as water-deficit and various heavy metals, the expression of miR166 was drastically increased [38,39]; Under aluminum stress, gma-miR166 of Al-tolerant soybeans were significantly down-regulated [40]. Therefore, miR166 may regulate biological processes to enable plant cross adaptation to various stresses [41]. In Arabidopsis, miR165/166 regulates the expression of class-II HD-Zip genes which are necessary for the formation of lateral roots and vascular bundles [42]. Studies have shown that overexpression of miR166/165 inhibited class-III HD-Zip genes, thereby inhibiting the root system of Arabidopsis [43]. In this study, after 12h and 8d of B stress, miR166 was highly expressed. This result further confirms that B stress can affect the growth and division of soybean root cells. At 12h of B stress, gma-miR166j-5p showed a downward trend. With prolonged exposure to B stress, at 8d, the expression of gma-miR166j-3p in the miR166 family was observed to be extremely high and significantly down-regulated. This indicates that the root system of MK9 was subjected to inhibition of growth due to B stress, and MK9 exhibited certain resistance to B stress through self-regulation.

The change of nutrient concentration in the external environment will destroy the osmotic potential of plant roots. The GEMs of miR4996 were target to the NADP-dependent male enzyme (NADP-ME) gene. NADP-ME is one of the key enzymes in malic acid metabolism and playing an essential role in maintaining cell osmotic potential, stabilizing cytoplasmic pH, and balancing ion absorption [44–46].

This is a key reactive oxygen species (ROS) that can seriously damage cells. MiR4996 targets control calcium dependent protein kinase (CDPK) (Fig 9). miR398 targets two closely linked to Cu/Zn superoxide dismutase (CSD1 and CSD2) that can detoxify superoxide radicals [47]. The study of tobacco under the Cu conditions, showed that the expression of miR398 increased notably, and compared with the wild-type, transgenic lines accumulated higher levels of SOD activity [47]. In this study, SOD, POD, and CAT activity in soybean were extensively enhanced under B stress at 12h and 8d, while the expression of gma-miR398d increased sharply, and the change trend of protective enzyme was consistent with the copper stress in tobacco. This indicates that the activity of protecting oxygen in soybean was increased through the up-regulation of miR398d, which made soybean adapt to the B deficiency environment.

In wild soybeans, miR169 was up-regulated after Al treatment [25], whereas in Al-sensitive soybeans, it did not change significantly. However, the oxidoreductase activity was drastically enhanced [40]. In this study, miR169 showed no significant difference at 12h, but gma-miR169j-3p was extensively up-regulated at 8d, which was similar to wild soybeans. In our experiment, B stress would affect the miRNA related to redox and other metabolic reactions, further affecting the activity of protective enzymes in plants, and thus inhibiting the growth and development of the soybeans.

**Fig 10:**
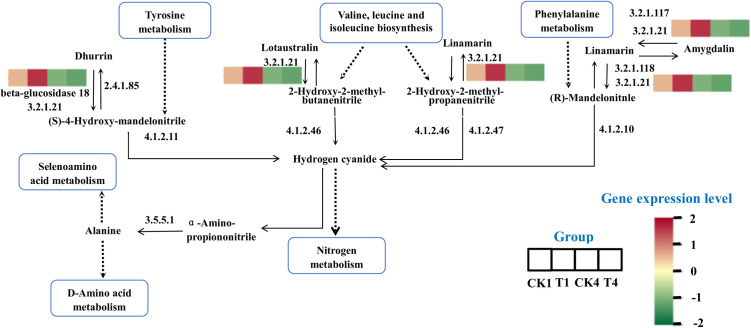
Cyanoamino acid metabolism.

In this study, we found that miR408c-5p targeted the gene controlling β-glucosidase 18 (Fig 9). The expression level of β-glucosidase 18 (E3.2.1.21) gene was notably down-regulated by miR408c-5p. The multiple changes in the expression level of this gene were significant in CK4_ VS_T4. It indicated that the effect of B deficiency on this gene was significant. Based on transcriptomics data, this gene played a role in various metabolic pathways, such as cyanoamino acid metabolism (Fig 10), starch and sucrose metabolism, degradation of flavonoids and biosynthesis of variable plant secondary metabolites. In this experiment, cyanogenic amino acid metabolism, glycine, serine and threonine metabolism, and phenylalanine metabolism play important roles in boron stress. Among them, cyanogenic amino acid metabolism was the most significantly enriched metabolic pathway in the results of transcriptomics data (CK4_VS_T4) [9]. The synthesis of a wide range of metabolic and intracellular signaling molecules and proteins, as well as the formation of biofilms, are related to the metabolism of Cyanoamino acid metabolism [48]. This evidence suggested that this pathway may have a significant role in B stress.

## Conclusions

In the study of B stress in soybeans at different time points, it was found that the plant was most affected at 8 days, and the root system was more susceptible to B stress than the leaves. High-throughput miRNA sequencing was performed on soybean roots after 12 hours and 8 days of stress, respectively; and 55 DEMs were identified in CK1_vs_T1, CK4_vs_T4 and T1_vs_T4. Among the identified differential miRNAs, 43 miRNAs were predicted to have 695 target genes. These miRNAs were analyzed and found to be probably closely related to boron stress. Comprehensive mRNA data found that they were mainly involved in chlorophyll synthesis, soybean root growth and development, root osmotic potential, oxidation-reduction activity and protective enzyme activity, and cyanoamino acid metabolism, etc., by affecting its objective genes to inhibited soybean seedling growth. Most importantly, we analyzed, for the first time, the regulatory mechanism of cyanoamino acid metabolism inhibiting soybean growth under B stress and analyzed the possible expression patterns. We hope to further investigate the response mechanism of soybeans to B stress. The research results provide valuable information for soybean B stress and new ideas for soybean molecular breeding.

## Supporting information

S1 Table Information on primers used in this study. (DOCX)
