# Supplementary material for: An integrated analysis of miRNA and mRNA expressions in soybean response to boron stress
Source: PLoS One. 2025 Jul 28;20(7):e0328882. doi: 10.1371/journal.pone.0328882 (PMC12303299; doi:10.1371/journal.pone.0328882)
Supplement: S1 Table — (DOCX) [file pone.0328882.s001.docx]

**Table S1.** Information on primers used in this study

| **Pri ID** | **Pri seq (5’ to 3’)** |
| --- | --- |
| gma-MIR5770a-F | CCGCAAAAGGGGAATAAGAT |
| gma-MIR5770a-R | TGGACAAGTGGCAACTGAAC |
| gma-MIR408c-F | AGAGCATGGATGGAGCTATCA |
| gma-MIR408c-R | AGTGCATGAGCAGAACCACA |
| gma-MIR390g-F | CGCCACGACACTCATGATTT |
| gma-MIR390g-R | CTCAGGATAGATAGCGCCAAAT |
| gma-MIR169j-F | GTAGCCAAGAATGACTTGCC |
| gma-MIR169j-R | GTAGCCAAGAACAACTCGTC |
| gma-MiR171q-F | GAACTTGAGATATTGGTGCGGT |
| gma-MiR171q-R | GTGATATTGGCACGGCTCAA |
| gma-MIR156a (internal reference)-F | CACCAGATTGAGAGAGGCTGA |
| gma-MIR156a (internal reference)-R | ACGCACCCGCAATTGTAT |
